# Acanthocephalans from fishes and amphibians in Vietnam, with descriptions of five new species

**DOI:** 10.1051/parasite/2014052

**Published:** 2014-10-22

**Authors:** Omar Mohamed Amin, Richard Anderson Heckmann, Nguyen Van Ha

**Affiliations:** 1 Institute of Parasitic Diseases 11445 E. Via Linda 2-419 Scottsdale Arizona 85259 USA; 2 Department of Biology, Brigham Young University 401 WIDB Provo UT 84602 USA; 3 Department of Parasitology, Institute of Ecology and Biological Resources (IEBR), Vietnam Academy of Science and Technology 18 Hoang Quoc Viet Cau Giay Hanoi Vietnam

**Keywords:** Acanthocephala, Vietnam, Fish, Amphibian

## Abstract

Eight species of acanthocephalans are reported, and five are new. Specimens of *Neoechinorhynchus* (*Hebesoma*) *manubrianus* Amin, Ha & Ha, 2011 were similar to the original description. *Neoechinorhynchus* (*Hebesoma*) *spiramuscularis* n. sp. (Neoechinorhynchidae), from *Xenocypris davidi*, has a unique proboscis receptacle wrapped in a spiral muscular layer, and an undulating flask-shaped lemnisci, as well as double para-receptacle structures. *Heterosentis mongcai* n. sp. (Arhythmacanthidae), from *Acreichthys* sp., has a small fusiform trunk with an unarmed cone and anterior trunk spines, and a proboscis with two circles of rooted apical hooks and 3–4 circles of rooted posterior spines as well as a para-receptacle-like structure at the posterior end. The poorly known *Filisoma indicum* Van Cleave, 1928 is fully described and illustrated for the first time. *Acanthocephalus parallelcementglandatus* n. sp. (Echinorhynchidae), from *Clarias batrachus*, is distinguished from other species of *Acanthocephalus* by its small fusiform trunk and parallel tubular cement glands. *Pseudoacanthocephalus coniformis* n. sp. (Echinorhynchidae), from *Hylarana* sp., is distinguished from other species by having an anterior trunk collar and staggered prominent filiform cement glands, among other features. *Cathayacanthus spinitruncatus* n. sp. (Rhadinorhynchidae), from *Leiognathus equulus*, is distinguished from the only two known species of the genus by having a very long and slender proboscis with more than 50 hooks per row and a totally spined trunk. The generic diagnosis of *Cathayacanthus* Golvan, 1969 is emended. *Rhadinorhynchus johnstoni* Golvan, 1969 (Rhadinorhynchidae) perfectly fits the only complete description of that species from the Fiji Islands.

## Abstract (fr)

Huit espèces d’Acanthocéphales sont rapportées et cinq sont nouvelles. Les spécimens de *Neoechinorhynchus (Hebesoma) manubrianus* Amin, Ha, Ha & 2011 étaient semblables à la description originale. *Neoechinorhynchus (Hebesoma) spiramuscularis* n. sp. (Neoechinorhynchidae), de *Xenocypris davidi*, présente un réceptacle de proboscis unique enveloppé dans une couche musculaire spiralée, des lemnisques en forme de flasques ondulées et des structures para-réceptaculaires doubles. *Heterosentis mongcai* n. sp. (Arhythmacanthidae), de *Acreichthys* sp., a un petit tronc fusiforme avec un cône non épineux et des épines antérieures sur le tronc, un proboscis avec deux cercles de crochets apicaux avec racines, 3-4 cercles d’épines postérieures avec racines et une structure para-réceptaculaire à l’extrémité postérieure. *Filisoma indicum* Van Cleave, 1928, mal connu, est entièrement décrit et illustré pour la première fois. *Acanthocephalus parallelcementglandatus* n. sp. (Echinorhynchidae) de *Clarias batrachus*, se distingue des autres espèces d’*Acanthocephalus* par son petit tronc fusiforme et des glandes cémentaires tubulaires et parallèles. *Pseudoacanthocephalus coniformis* n. sp. (Echinorhynchidae), de *Hylarana* sp., se distingue des autres espèces par un collier sur le tronc antérieur et des étonnantes glandes cémentaires filiformes, entre autres caractéristiques. *Cathayacanthus spinitruncatus* n. sp. (Rhadinorhynchidae), de *Leiognathus equulus*, se distingue des deux seules espèces connues du genre par un proboscis très long et mince avec plus de cinquante crochets par ligne et un tronc totalement épineux. Le diagnostic générique de *Cathayacanthus* Golvan 1969, est émendé. *Rhadinorhynchus johnstoni* Golvan, 1969 (Rhadinorhynchidae) correspond parfaitement à la seule description complète de cette espèce, des Îles Fidji.

## Introduction

A number of acanthocephalan species from freshwater fishes and other vertebrates were previously described in Vietnam by Amin and Ha [3, 4] and Amin *et al*. [6, 9–11, 32]. Eleven species of acanthocephalans were collected from marine fishes off the eastern seaboard of Vietnam at Halong Bay in 2008 and 2009. Of these, 6 new species belonging to *Neoechinorhynchus* Stiles and Hassall, 1905, one new species of *Heterosentis* Van Cleave, 1931, and three new species of *Acanthocephalus* Koelrouther, 1771, *Gorgorhynchus* Chandler, 1934, and *Neorhadinorhynchus* Yamaguti, 1939 were recently described by Amin and Ha [4]. Two new species of *Rhadinorhynchus* Lühe, 1911 were described from marine fishes in the same bay [7]. Three other species of *Rhadinorhynchus* were previously reported from marine fishes in Vietnam [14]. The species reported in this presentation have not been previously encountered by us or by any other observer in Vietnam. The above contributions by Amin and collaborators, among others, as well as those listed in Arthur and Te [14] should be consulted for information about the current state of knowledge on the Acanthocephala of Vietnamese vertebrates. The present contribution will add significant information to this database.

## Materials and methods

Nine species of fishes and one amphibian were collected in five diverse habitats from distant locations in Vietnam between 2010 and 2013. The marine fishes *Nibea albiflora* (Richardson) and *Johnius carouna* (Cuvier) (Sciaenidae) were examined from the Pacific Ocean off the Cát Bà Islands, Halong Bay, Gulf of Tonkin (107°05′ E, 20°45′ N) in August and October, 2013. The marine fishes *Acreichthys* sp. (Monacanthidae) and *Epinephelus* sp. (Serranidae) were collected in the Pacific Ocean at Mong Cai District, Quang Ninh Province in the northeast corner of Vietnam north of Tonkin Bay (21°25′ N; 108°05′ E) on May 16, 2013. The freshwater fishes *Clarias batrachus* (Linn. 1758) (Clariidae) and *Xenocypris davidi* Bleeker, 1871 (Cyprinidae) were collected from the Ma River in the Ben En National Park in Thanh Hóa Province (19°37′ N; 105°31′ E) on April 25, 2010. The marine flying fish *Cypselurus hexazona* (Bleeker, 1853) (Exocoetidae) was collected in the Pacific Ocean at Quang Binh Province along Vietnam’s north central coast south of Tonkin Bay (17°31′ N; 106°39′ E) on April 24, 2013. Another marine fish, *Scatophagus argus* (Linn., 1766) (Scatophagidae), was also collected in the Pacific Ocean at Kiên Giang Province in the Mekong Delta region of southern Vietnam (10°21′ N; 104°26′ E) in October, 2012. The common pony fish, *Leiognathus equulus* Forsskål (Leiognathidae), was collected south of the Tonkin Gulf in the Hue City area, Thua Thien Hue Province, Central Vietnam (16°43′ N; 107°45′ E) in August, 2013. The frog *Hylarana* sp. (Ranidae) was collected in Bidoup Nui Ba National Park, Lâm Dông Province (12°18′ N; 108°40′ E) on June 6, 2013.

Live specimens were kept in tap water for a few hours until proboscides were everted then fixed in 70% ethanol. Specimens were then shipped to Parasitology Center, Inc., Arizona. These specimens were stained in Mayer’s acid carmine, destained in 4% hydrochloric acid in 70% ethanol, dehydrated in ascending concentrations of ethanol (70%, 80%, 90% twice, 100%), and cleared in 100% xylene, then in 50% Canada balsam and 50% xylene; each step for 24 hr. Whole worms were then mounted in Canada balsam. Measurements are in micrometers, unless otherwise noted; the range is followed by the mean values between parentheses. Width measurements represent maximum width. Trunk length does not include the proboscis, neck or bursa.

For scanning electron microscopy (SEM) studies, two specimens of *Cathayacanthus spinitruncatus* n. sp. previously fixed in 70% ethanol were placed in critical-point drying baskets and dehydrated using ethanol series of 95% and 100% for at least 10 min per soak, followed by critical-point drying [23]. Samples were mounted on SEM sample mounts, gold/palladium- coated, and observed with a scanning electron microscope (XL30 ESEMFEG; FEI, Hillsboro, Oregon). The small numbers of the other species reported did not allow for additional SEM studies.

## Species found

Eight species in two classes, two orders, and five families of acanthocephalans were collected from six species of Vietnamese fishes and one species of amphibian between 2010 and 2013 as follows:

Class Eoacanthocephala Van Cleave, 1936

 Order Neoechinorhynchida Southwell and Macfie, 1925

  Family Neoechinorhynchidae (Ward, 1917) Van Cleave, 1928

   *Neoechinorhynchus* (*Hebesoma*) *manubrianus* Amin, Ha and Ha, 2011

   *Neoechinorhynchus* (*Hebesoma*) *spiramuscularis* n. sp.

Class Palaeacanthocephala Meyer, 1931

Order Echinorhynchida Southwell and Macfie, 1925

  Family Arhythmacanthidae Yamaguti, 1935

   *Heterosentis mongcai* n. sp.

  Family Cavisomidae Meyer, 1932

   *Filisoma indicum* Van Cleave, 1928

  Family Echinorhynchidae Cobbold, 1876

   *Acanthocephalus parallelcementglandatus* n. sp.

   *Pseudoacanthocephalus coniformis* n. sp.

  Family Rhadionorhynchidae Lühe, 1912

   *Cathayacanthus spinitruncatus* n. sp.

   *Rhadinorhynchus johnstoni* Golvan, 1969

## *Neoechinorhynchus* (*Hebesoma*) *manubrianus* Amin, Ha & Ha, 2011

Five specimens (three males, two females) of *N.* (*H.*) *manubrianus* Amin, Ha and Ha, 2011 were collected from the marine fish *Nibea albiflora* (Richardson) in August, 2013, and one male specimen was collected from *Johnius carouna* (Cuvier) (Sciaenidae) in October, 2013 in the Pacific Ocean off the Cat Ba Islands, Halong Bay, Gulf of Tonkin (107° 05′ E, 20°45′ N). The specimens (HWML collection nos. 49911, 49912) were similar to the original description [12].

## *Neoechinorhynchus* (*Hebesoma*) *spiramuscularis* n. sp.

(Figs. 1–7)

**Figures 1–7:**
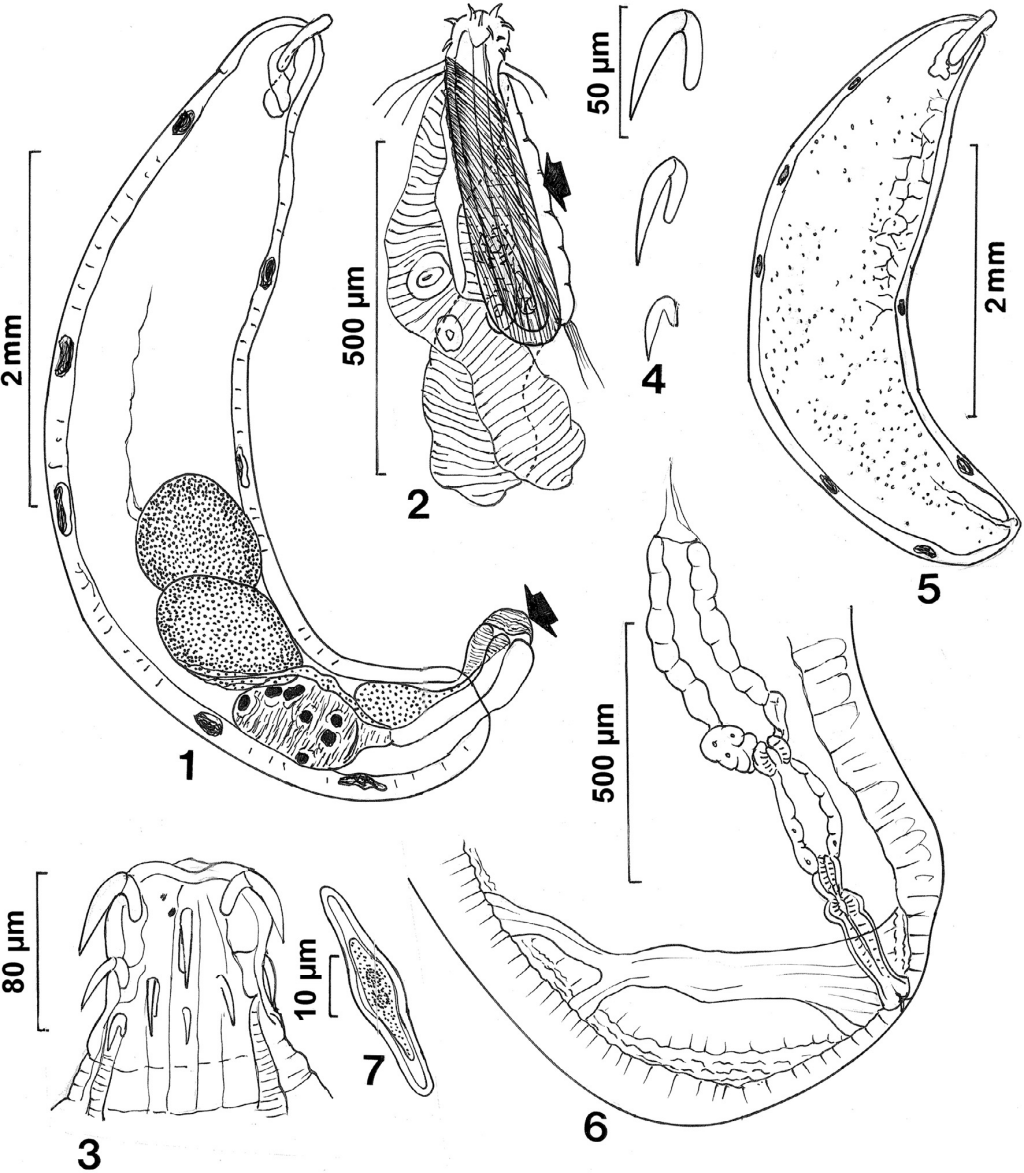
*Neoechinorhynchus* (*Hebesoma*) *spiramuscularis* n. sp. from *Xenocypris davidi.* 1. Holotype male. Note the copulatory plug capping the bursa (arrow). 2. Anterior end of specimens in Figure 1 showing the spiral muscles wrapping around the proboscis receptacle, the undulating lemnisci, and the para-receptacle structure (arrow). 3. The proboscis of a paratype female. Note the two small giant nuclei near the apex. 4. A row of proboscis hooks. 5. Allotype female. Note a small part of the reticular secondary lacunar branches. 6. The reproductive system of a paratype female. Note the prominent dorso-ventral girdle with dorsal branching characteristic of this species. 7. Egg.

urn:lsid:zoobank.org:act:4C0E1728-574B-4CD3-93FD-E2200C6908DB

Type host: *Xenocypris davidi* (Cyprinidae).

Type locality: Ma River in Ben En National Park in Thanh Hóa Province (190°37′ N; 105°31′ E), Vietnam.

Type material: HWML collection no. 49913 (holotype male and allotype female on one slide), no. 49914 (paratypes, two slides).

Etymology: The species is named after its unique spiral muscular coat enveloping the proboscis receptacle.

Twenty-two specimens of this species (11 males and 11 females) were collected from 1 of 10 specimens of *Xenocypris davidi* (Cyprinidae) from a tributary of the Ma River in Ben En National Park in Thanh Hóa Province (19°37′ N; 105°31′ E) on April 25, 2010. This subtropical freshwater fish is found in China where it is also reported in brackish waters [25]. The collected worms included underdeveloped immature males (5) and females (4).

### Description

*General*: With characters of the genus *Neoechinorhynchus* and the subgenus *Hebesoma* (Neoechinorhynchidae) as described by Amin (2002). Small fusiform worms, wider at middle, with arched trunk usually posteriorly, gradually tapering at both ends. Body wall with five (rarely four) dorsal and two (rarely three) ventral giant hypodermal nuclei (Figs. 1, 5). Secondary branches of lacunar system with reticular anastomoses (Fig. 5). Proboscis small, rectangular, slightly wider than long, with two small giant nuclei near apex on same surface. Lateral hooks in anterior ring slightly more posterior than dorsal and ventral hooks (Fig. 3). Middle hooks slightly smaller than and closer to anterior than to posterior hooks. Posterior hooks smallest. All hooks rooted; roots simple, slender, blade-like, shorter than hooks, directed posteriorly (Figs. 3, 4). Neck prominent, markedly wider posteriorly. Proboscis receptacle single-walled, much longer than proboscis, wrapped in prominent spiral muscular layer fanning from insertion area at anterior ventral side of receptacle. Dorsal and ventral para-receptacle structures (PRS) well developed (Fig. 2, arrow). Dorsal PRS often masked by lemnisci often positioned dorsal to receptacle. Lemnisci sub-equal, much longer than receptacle, narrow anteriorly but becoming heavily undulating and larger posteriorly, each with two prominent giant nuclei (Fig. 2).

*Male* (*based on six mature males with sperm*): trunk 4.65–8.00 (6.01) mm long by 0.87–1.90 (1.38) mm wide anteriorly. Proboscis 67–80 (74) long by 80–97 (92) wide. Proboscis hook length from anterior 37–42 (40), 37–40 (38), 22–25 (23), respectively. Neck 45–50 (48) long by 125–145 (136) wide posteriorly. Proboscis receptacle 416–520 (545) long by 98–145 (123) wide. Para-receptacle structure 281–343 (313) long. Shorter lemniscus 0.45–1.06 (0.79) mm long by 0.11–0.36 (0.20) mm wide posteriorly. Longer lemniscus 0.68–1.30 (0.99) mm long by 0.15–0.28 (0.21) mm wide posteriorly. Reproductive system in posterior half of trunk; testes partially overlap (Fig. 1). Anterior testis 0.60–1.42 (0.93) mm long by 0.45–1.22 (0.77) wide. Posterior testis 0.70–1.62 (1.02) mm long by 0.47–1.15 (0.71) mm wide. Cement gland 0.47–1.25 (0.87) mm long by 0.35–1.20 (0.65) mm wide, with 6–8 giant nuclei and small conical cement reservoir posteriorly. Common sperm duct inflated anteriorly, extending with Saefftigen’s pouch to jointly empty into bursa. Common sperm duct 416–750 (591) long by 125–300 (250) wide. Saefftigen’s pouch 728–759 (745) long by 166–187 (173) wide. Gonopore terminal. Bursa 600–728 (668) long by 375–425 (401) wide, occasionally capped with copulatory plug (Fig 1, arrow).

*Female* (*based on seven mature females with eggs at various stages of development*): trunk 5.12–10.57 (7.95) mm long by 1.25–2.25 (1.78) mm wide anteriorly. Proboscis 67–104 (81) long by 94–114 (104) wide. Proboscis hook length from anterior 42–52 (45), 37–47 (40), 25–31 (26), respectively. Neck 55–94 (76) long by 112–166 (145) wide posteriorly. Proboscis receptacle 416–572 (474) long by 114–146 (131) wide. Para-receptacle structure 287–426 (349) long. Shorter lemniscus 0.60–1.50 (0.95) long by 0.17–0.33 (0.26) wide posteriorly. Longer lemniscus 0.69–2.53 (1.29) long by 0.25–0.47 (0.31) wide posteriorly. All parts of reproductive system well developed, 0.94–1.25 (1.08) long (11–15% of trunk length). Triangulate vestibular muscles inserting base at ventral body wall just anterior to sub-ventral gonopore overlapping vagina then extending dorso-ventrally before branching and attaching to opposite dorsal body wall (Fig. 6). Eggs fusiform with polar prolongation of fertilization membrane, 30–37 (33) long by 6–10 (9) wide (Fig. 7).

### Remarks

The new species is unique among all 14 species of the subgenus *Hebesoma* Van Cleave, 1928 listed in Amin [2] by three traits: (1) the muscle layer wrapped around the proboscis receptacle, (2) the undulating flask-shaped lemnisci that are strikingly much broader posteriorly than anteriorly, and (3) the prominent dorso-ventral vestibular muscle at the posterior end of females overlapping the vagina. Actually, none of the other species of the other subgenus, *Neoechinorhynchus* Hamann, 1892, or the unassigned species [2] shares these three characteristics. Of the species of *Hebesoma*, three bear only one superficial similarity to the new species in having a small fusiform trunk shape: (1) *N*. (*H*.) *manasbalensis* Kaw, 1951 from Kashmir, which has a larger proboscis, smaller proboscis receptacle and 8–10 dorsal giant hypodermal nuclei, (2) *N*. (*H*.) *pungitius* Dechtiar, 1971 from Canada has a smaller trunk, equatorial testes that fill the body cavity, terminal female gonopore and ovoid eggs, and (3) *N*. (*H*.) *violentus* (Van Cleave, 1928) Salgado-Maldonado, 1978 from China that has a non-arched body, equatorial testes, terminal female gonopore and posteriorly constricted male trunk. All the other 11 species of *Hebesoma* have a cylindrical trunk, tubular lemnisci, no spiral muscular layer around the receptacle, and no vestibular muscles at the posterior end of females: *N*. (*H*.) *agilis* (Rudolphi, 1819) Van Cleave, 1916; *N*. (*H*.) *anguillum* El-Damarani, 2001; *N*. (*H*.) *carinatus* Buckner and Buckner, 1993; *N*. (*H*.) *chrysemydis* Cable and Hopp, 1954; *N*. (*H*.) *didelphis* Amin, 2001; *N*. (*H*.) *doryphorus* Van Cleave and Bangham, 1949; *N*. (*H*.) *idahoensis* Amin, and Heckmann, 1992; *N*. (*H*.) *kallarensis* George and Nadakal, 1978; *N*. (*H*.) *lingulatus* Nickol and Ernst, 1987; *N*. (*H*.) *manubrianus* Amin, Ha and Ha, 2011; *N.* (*H*.) *rostratus* Amin and Bullock, 1998.

Additionally, the new species is distinguished by having a PRS which has not been described in any of the other species of *Hebesoma* listed above. The PRS is a primitive contractile structure that reportedly uses hydrostatic pressure to effect the eversion of the proboscis in eoacanthocephalans that have only a weak single-walled proboscis receptacle (Amin et al., 2007). The PRS was first reported in *Neoechinorhynchus* (*N*.) *qatarensis* Amin, Saoud, and Alkuwari, 2002 and its structural-functional relationship described in the same species [8]. To date, it is known in a very few species of *Neoechinorhynchus* Stiles and Hassall, 1905 and of the subgenus *Acanthosentis* Verma and Datta, 1929. These include *Neoechinorhynchus* (*N*.) *golvani* Salgado-Maldonado, 1978, *Neoechinorhynchus ampullata* Amin, Ha and Ha, 2011, and *Neoechinorhynchus* (*N*.) *ascus* Amin, Ha and Ha, 2011 as well as *Acanthogyrus* (*Acanthosentis*) *parareceptaclis* Amin, 2005, and *Acanthogyrus* (*Acanthosentis*) *barmeshoori* Amin, Gholami, Akhlaghi and Heckmann, 2013.

## *Heterosentis mongcai* n. sp.

(Figs. 8–11)

**Figures 8–14:**
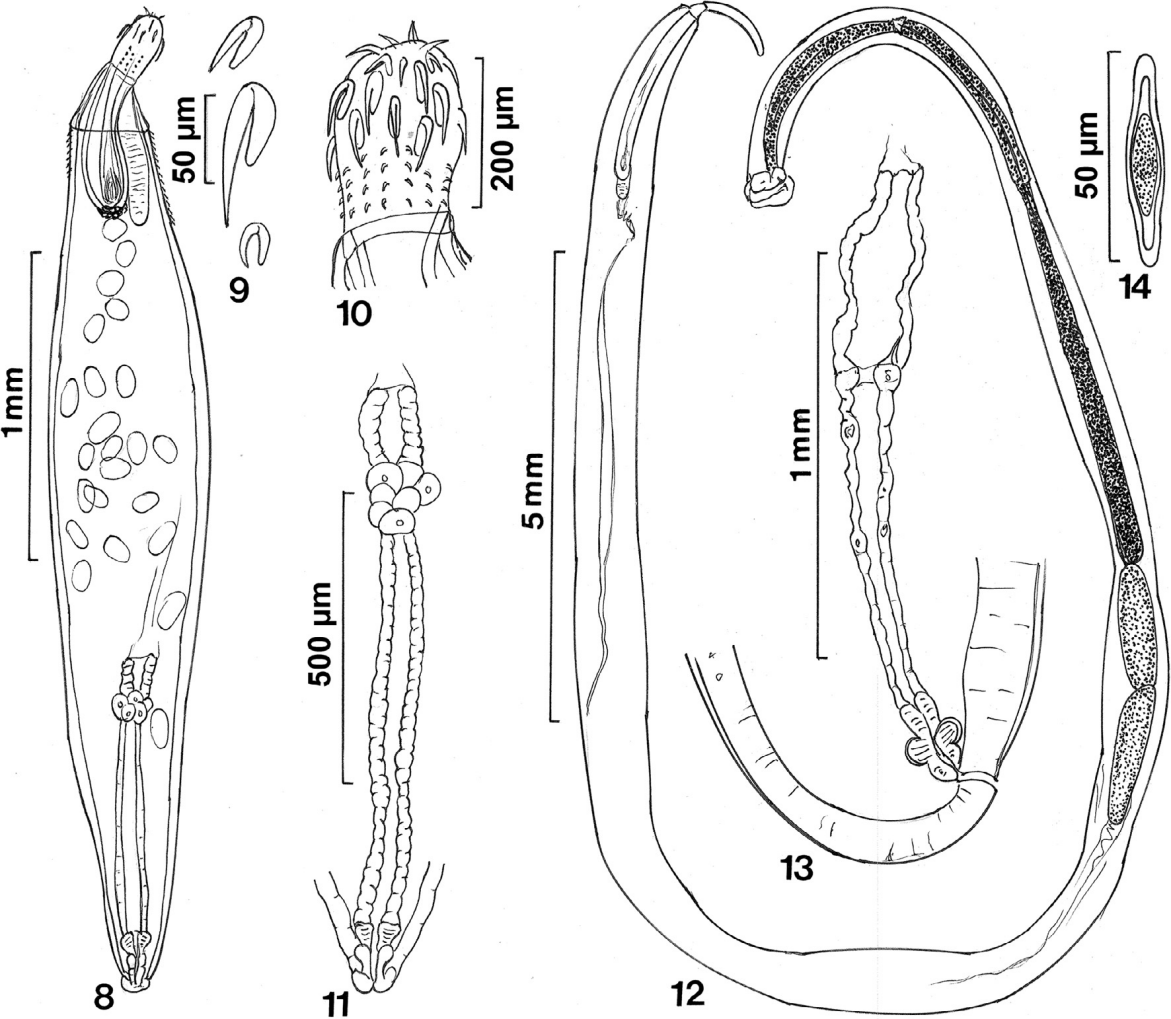
8–11: *Heterosentis mongcai* n. sp. from *Acreichthys* sp. 8. Holotype female. Note the nuclei at the posterior end of the proboscis receptacle, the anterior trunk cone, and the distribution of trunk spines. 9. A row of proboscis hooks. 10. Proboscis. 11. Detail of the reproductive system. 12–14: *Filisoma indicum* Van Cleave, 1928 from *Scatophagus argus.* 12. A male showing the distribution of various reproductive system structures. 13. Detail of the female reproductive system. 14. Egg.

urn:lsid:zoobank.org:act:CD733F2D-5F69-40E4-89F8-2E36A8A02BB4

Type host: Filefish *Acreichthys* sp. (Monacanthidae).

Other host: Grouper *Epinephelus* sp. (Serranidae)

Type locality: Pacific Ocean at Mong Cai District, Quang Ninh Province, northeast corner of Vietnam north of Tonkin Bay (21°25′ N; 108°05′ E), Vietnam.

Type material: HWML collection no. 49915 (holotype female)

Etymology: The species was named for the collecting site: the Pacific Ocean at Mong Cai District.

Two female specimens of this species were collected from two species of marine fishes: one of two specimens of the filefish *Acreichthys* sp. (Monacanthidae) and one of six examined specimens of the grouper *Epinephelus* sp. (Serranidae) in the Pacific Ocean at Mong Cai District, Quang Ninh Province in the northeast corner of Vietnam north of Tonkin Bay (21°25′ N; 108°05′ E) on May 16, 2013. *Acreichthys* is a genus of filefishes native to the Indian and Pacific oceans and includes three recognized species. Groupers are large sea fishes with 99 recognized species in the genus *Epinephelus* [17].

### Description

Female (based on two specimens in the ovarian ball stage); with characters of the genus *Heterosentis* (Arhythmacanthidae): Small fusiform worms, 2.52–2.97 (2.74) mm long by 0.52–0.59 (0.55) mm wide at middle, with unarmed anterior trunk cone, 198 long by 244–249 (246) wide posteriorly. Anterior 5–8 long random trunk spines extend short distance, 150–160 (155) past level of proboscis receptacle ventrally but a shorter distance, 50–55 (52) dorsally (Fig. 8). Proboscis 208–229 (218) long by 146–156 (151) wide anteriorly, with 14 rows of 2 anterior hooks and 3–4 posterior spines each. Anterior proboscis globular with 1 anterior circle of small slightly curved apical hooks, 40–42 (41) long and second circle of larger, more sharply curved sub-apical hooks in two tiers: 80–95 (88) long anteriorly and 70–92 (78) long posteriorly. Posterior proboscis cylindrical with small curved spines (Fig. 9) gradually decreasing in size posteriorly, measuring from anterior 22–27 (25), 20–25 (22), 17–19–(18), 10–15 (14) in length (Fig. 10). All hooks and spines with slender but prominent roots. Roots of anterior hooks simple, directed posteriorly, about half as long as blades and curved in same direction. Roots of posterior spines somewhat shorter than blades and not curved (Fig. 9). Neck unremarkable. Proboscis receptacle double-walled, about twice as long as proboscis, 447–478 (462) long by 161–177 (169) wide, with large cephalic ganglion at its base. Posterior end of receptacle wall nucleated. Lemnisci digitiform, equal, slightly longer than receptacle (Fig. 8), 624–629 (626) long by 62–65 (63) wide. Reproductive system 1.09–1.15 (1.12) mm long (41% of trunk length), with prominent long uterus, short uterine bell, well-defined vagina, and terminal gonopore (Fig. 11). Eggs not available.

### Remarks

Amin et al. [13] described *Heterosentis holospinus* Amin, Heckmann and Ha, 2011 from the striped eel catfish, *Plotosus lineatus* (Thunberg, 1787), from the Tonkin Gulf, Halong Bay, Vietnam, and provided a key to all 15 known species [2]. Another species, *Heterosentis mongcai* n. sp., was found in two other fish species from the same waters and reported herein; an interesting correlation. There are now four known species of *Heterosentis* with an anterior trunk cone: *Heterosentyis holospinus* and *Heterosentis mongcai* from Vietnam, *Heterosentis plotosi* Yamaguti, 1935 from Japan, and *Heterosentis septacanthus* (Sita, 1949) Golvan, 1969 from India. The three other species differ from the new species as follows. The trunk spines of *H. holospinus* cover the whole trunk except the anterior cone. In the two other species, the anterior trunk cone is covered with spines and the posterior wall of the proboscis receptacle is not nucleated. Additionally, *Heterosentis plotosi* has four giant nucleated muscle cells in the trunk and *Heterosentis septacantus* has a cylindrical trunk; the trunk of *Heterosentis mongcai* is fusiform.

## *Filisoma indicum* Van Cleave, 1928

(Figs. 12–14)

Host: Spotted scat, *Scatophagus argus* (Scatophagidae)

Locality: Pacific Ocean at Kiên Giang Province in the Mekong Delta region of southern Vietnam (10°21′ N; 104°26′ E), Vietnam.

Voucher specimens: HWML collection no. 49916 (one slide).

Six specimens of this species (three males and three females including one juvenile) were collected from 1 of 5 specimens of the spotted scat, *Scatophagus argus* (Scatophagidae), in the Pacific Ocean at Kiên Giang Province in the Mekong Delta region of southern Vietnam (10°21′ N; 104°26′ E) in October, 2012. This tropical marine fish is also found in brackish reef-associated and fresh waters (Reide, 2004) of the Indo-Pacific region [24]; it feeds on worms, crustaceans, insects, and plant matter [26].

### Description of our Vietnamese specimens

*General*: With characters of the genus *Filisoma* (Cavisomatidae). Trunk unarmed, long, uniformly cylindrical (Fig. 12). Body wall with many scattered nucleated cells. Shared structures larger in females than in males. Proboscis long and cylindrical with short neck and 14–15 longitudinal rows of 24–28 (26) hooks each. Sub-apical 5–11 hooks in two ventral rows stout. Largest hooks near middle, gradually decrease in size anteriorly, and posteriorly. Posterior hooks smallest. All hooks rooted. Hook roots simple, directed posteriorly, shorter than blades, almost plug-like in spine-like posteriormost hooks. Basal spines not arranged in a ring. Double-walled proboscis receptacle about twice as long as proboscis with cephalic ganglion at its posterior end. Lemnisci digitiform, barely equal, about as long as proboscis receptacle (Fig. 12).

*Male* (based on three mature adults with sperm): trunk 14.75–30.00 (21.42) mm long by 0.25–0.77 (0.46) mm wide. Proboscis 832–936 (884) long by 93–104 (99) wide. Length of proboscis hooks near anterior, middle, and posterior end 30, 37, 20 on dorsal side; 30, 32, 22 on ventral side. Length of proboscis hook roots near anterior, middle, and posterior end 15, 20, 12 on dorsal side; 25, 25, 15 on ventral side. Proboscis receptacle 0.83–2.18 (1.68) mm long by 0.17–0.21 (0.19) mm wide. Lemnisci 0.91–2.24 (1.58) mm long by 0.075–0.21(0.14) mm wide. Reproductive system in posterior end of trunk with post-equatorial elongate testes (Fig. 12). Anterior testis 0.88–1.55 (1.26) mm long by 0.19–0.40 (0.32) mm wide. Posterior testis 0.83–1.25 (1.08) mm long by 0.21–0.37 (0.31) mm wide. Four tubular multinucleated cement glands 1.25–4.57 (3.51) mm long by 0.14–0.30 (0.20) mm wide. Saefftigen’s pouch overlapping posterior part of cement glands 780–936 (849) long by 125–270 (194) wide. Gonopore terminal. Everted bursa not available.

*Female* (based on two mature adults with eggs): trunk 42.00–72.50 (57.2) mm long by 0.82–1.12 (0.97) mm wide. Proboscis 1.14–1.22 (1.18) mm long by 0.14 wide. Length of proboscis hooks near anterior, middle, and posterior end 30–32, 30–37, 21–30 on dorsal side; 30–36, 29–40, 18–30 on ventral side. Length of proboscis hook roots near anterior, middle, and posterior end 16-25-15 on dorsal side; 25-27-19 on ventral side. Proboscis receptacle 1.43–2.39 (1.18) mm long by 0.22–0.29 (0.25) mm wide. Reproductive system 1.5 mm long (2% of trunk length), thick-walled with long uterus, well-developed vagina and uterine bell but no visible uterine bell glands. Gonopore subterminal without papillae (Fig. 13). Eggs elliptical with polar prolongation of fertilization membrane 50–55 (53) long by 12–16 (14) wide (Fig. 14).

### Remarks

Amin [2] listed 14 species of *Filisoma* Van Cleave, 1928 and acknowledged the synonymy of *Filisoma hoogliensis* Datta and Soota, 1962 with *F. indicum* first proposed by Gupta and Jain [20] and accepted by Amin and Nahhas [5]. Gupta and Jain’s [20] own “*F. indicum*” must be another species as it has 17–18 proboscis hook rows with hooks reaching as large as 40–51 by 10–19. We concur with Yamaguti [37], Golvan [19] and Gupta and Jain [20] in accepting Yamaguti’s [36] *F. indicum* as genuine despite his counting of six cement glands instead of the usual four. The orifice of the female gonopore in our and Yamaguti’s [36] specimens lacked papillae, in contrast to Van Cleave’s [33] description.

Van Cleave [33] and Datta and Soota [16] counted 14 and 12–14 proboscis hook rows each with 24 and 24–28 hooks, respectively. They provided inadequate short descriptions and only illustrated the proboscis, that was incomplete in the latter authors’ account. Our specimens from Vietnam had 14–15 (usually 14) hook rows with 24–28 hooks each. Features of our specimens from Vietnam were most similar to those described by Yamaguti [36], with some variations. Yamaguti’s [36] specimens had a relatively shorter trunk (28–34 mm in males, 38–56 mm in females) and proboscis (0.6–0.8 mm), 14–17 proboscis hook rows, each with 20–24 hooks, and 6 (?) cement glands.

Yamaguti’s [36] detailed description included illustration of the posterior end of a male and female showing a barely discernible female reproductive system and the posterior part of a male reproductive system. These structures and the well-illustrated proboscis in Van Cleave (1928, Fig. 3) are complemented by our illustrations of a whole male, showing the relationships between the proboscis, receptacle, lemnisci and reproductive system (Fig. 12), a recognizable female reproductive system (Fig. 13), and an egg (Fig. 14).

All records of *F. indicum* were reported from the same fish species, *S. argus*, in diverse habitats. The spotted scat is adaptable to a wide range of salinity and can be found in marine, brackish reef-associated or fresh waters [30] of the Indo-Pacific region [24], which explains the wide geographical distribution of *F. indicum*. It was found in fresh waters of Chilka Lake, India [33], River Hooghly, Calcutta, India [16], and the marine waters around Celebes (Sulawesi Island, Indonesia) [36] and Mekong Delta, Vietnam (this paper). The parasite is apparently able to extend its range of distribution along the geographical range of its host, which is also found in Kuwait to Fiji, north to southern Japan, south to New Caledonia, Samoa, Tonga, and the Society Islands [24]. It should not be surprising to discover *F. indicum* in the same host species from these other locations too.

## *Acanthocephalus parallelcementglandatus* n. sp.

(Figs. 15–18)

**Figures 15–22:**
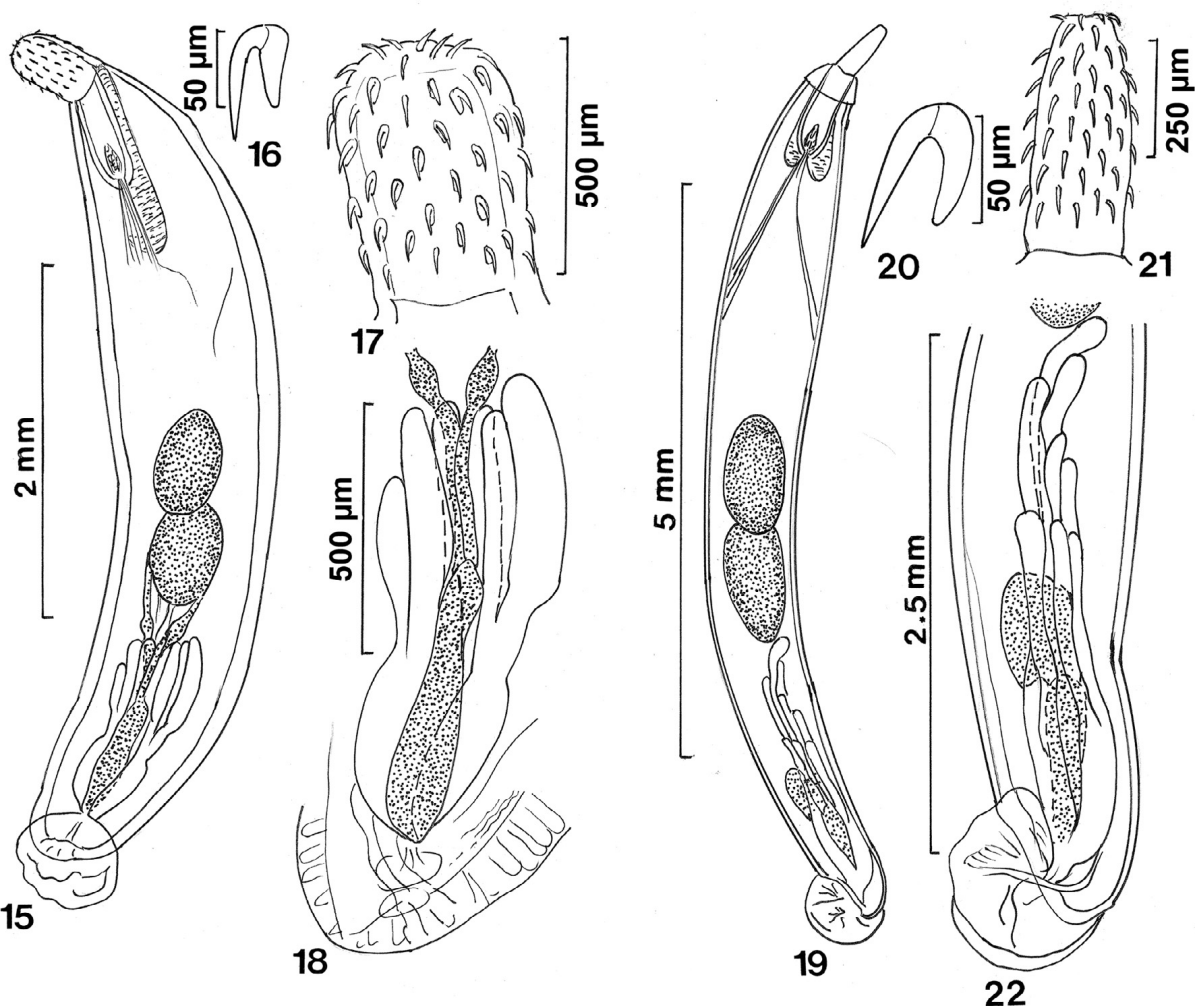
15–18: *Acanthocephalus parallelcementglandatus* n. sp. from *Clarias batrachus.* 15. Holotype male. Note the piercing of the incomplete outer proboscis receptacle posteriorly with retractor muscles. 16. A typical hook near the middle of the proboscis. 17. Proboscis. 18. Posterior part of the reproductive system showing detail of the parallel cement glands, common cement ducts, and sperm duct system (dotted). 19–22: *Pseudoacanthocephalus coniformis* n. sp. from *Hylarana* sp. 19. Holotype male. 20. A typical hook near the middle of the proboscis. 21. The proboscis with anterior apical end. 22. Detail of the posterior part of the reproductive system showing the staggered tubular cement glands and the common sperm duct system (dotted).

urn:lsid:zoobank.org:act:D7444FAE-E684-48F3-8667-47A63A368E53

Type host: Walking catfish *Clarias batrachus* (Clariidae)

Type locality: The Ma River, Ben En National Park, Thanh Hóa Province (19°37′ N; 105°31′ E), Vietnam.

Type specimen: HWML collection no. 49917 (holotype male).

Etymology: The new species is named for its characteristic parallel tubular cement glands.

One mature male specimen was collected from 1 of 15 specimens of the walking catfish *Clarias batrachus* (Clariidae) in the Ma River, Ben En National Park, Thanh Hóa Province (19°37′ N; 105°31′ E) on April 25, 2010. This omnivorous Asian freshwater fish feeds voraciously and rapidly on smaller fish, mollusks, and other invertebrates as well as on detritus and aquatic weeds [18]. Yet, only 1 of 15 examined fish had only 1 acanthocephalan.

### Description

*Male* (based on one mature specimen with sperm): with characters of the genus *Acanthocephalus* (Echinorhynchidae). Trunk aspinose, fusiform, small (4.30 mm long by 0.95 wide at middle), gradually tapering toward both ends. Body wall thinnest anteriorly and ventrally; thickness anteriorly 52 ventrally, 104 dorsally, and posteriorly 104 ventrally, 156 dorsally (Fig. 15). Proboscis short (447 long by 291 wide), cylindroid with 18 rows of similarly rooted 5 hooks each (Figs. 16, 17). Apical and basal hooks smallest and most slender. Ventral hooks larger than dorsal hooks. Hook roots prominent, lanceolate, relatively shorter than blades. Length of dorsal hooks (and roots) from anterior 67 (45), 75 (52), 75 (52), 62 (50), 60 (45). Length of ventral hooks (and roots) from anterior 71 (50), 80 (55), 77 (52), 75 (55), 70 (50). Neck 208 long by 374 wide at base. Proboscis receptacle double-walled but outer wall not continuous posteriorly where retractor muscles pass, 676 long by 270 wide, with prominent cephalic ganglion at its base (Fig. 15). Lemnisci markedly longer than receptacle, pyriform, equal, 946 long by 198 wide posteriorly. Reproductive system in posterior 3/5 of trunk with contiguous near post-equatorial testis. Anterior testis 582 long by 333 wide. Posterior testis bigger, 603 long by 437 wide. Cement glands, short distance from posterior testis, tubular, in two tight clusters of three glands each, 332–572 (447) long by 104–135 (122) wide, each cluster draining into one cement duct, 208–260 long. Each of two sets of cement gland ducts join into one common cement gland duct, 416–458 (437) long by 146–156 (151) wide. Longer sperm duct from anterior testis 749 long by 73 wide at swelling. Shorter sperm duct from posterior testis 645 long by 83 wide at swelling. Common sperm duct 728 long by 146 wide posteriorly positioned between two sets of cement glands. Saefftigen’s pouch 738 long by 156 wide, overlapping common sperm duct (Fig. 18). Gonopore terminal. Bursa without special features, 572 long by 624 wide (Fig. 15).

### Remarks

Amin [2] listed 53 species in the genus *Acanthocephalus* Koelreuther, 1771, of which 26 species are known in Asia and associated regions. Of these 26 species, 16 species are found in mainland Asia, 8 in Japan, and 2 in Australia. *Acanthocephalus parallelcementglandatus* n. sp. is separated from all 26 species by having (1) a small fusiform body, and (2) tubular parallel cement glands in 2 close clusters of 3 glands each. Each of the other 25 species has a cylindrical body (except *Acanthocephalus criniae* Snow, 1971as per Lesley Warner, pers. comm., who examined type material at the Tasmania Museum) and round, ovoid, or pyriform cement glands in various arrangements. In *A. criniae*, the trunk is flattened dorsoventrally, the proboscis has 11–16 longitudinal hook rows, the testes are equatorial, the cement glands are pyriform in a rosette pattern with long common ducts, and the genital pore is subterminal in both sexes. The new species is also distinguished by having two sets of three very short cement gland ducts joining into two prominent common sperm gland ducts (reservoirs). A list of the 26 Asian, Japanese, and Australian species of *Acanthocephalus* referred to above follows:

***Acanthocephalus atratus*** Van Cleave, 1925 (*nec aratus*) [syn. *Acanthocephalus lucidus* (*fide* Harada 1935, *fide* Yamaguti1939)] in Japan.
***Acanthocephalus criniae*** Snow, 1971 in Australia.
***Acanthocephalus curtus*** (Achmerov and Dombrovskaja-Achmerova, 1941)Yamaguti, 1963 [syns. *Paracanthocephalus curtus* Achmerov and Dombrovskaja-Achmerova,1941; *Acanthocephalus amuriensis* Kostylew, 1941] in Siberia.
***Acanthocephalus echigoensis*** Fujita, 1920 [syns. *Acanthocephalus acerbus* Van Cleave, 1931; *A. aculeatus* Van Cleave, 1931 (*fide* Harada 1935); *A. onchorhynchi* Fujita, 1920] in Japan.
***Acanthocephalus elongatus*** Van Cleave, 1937 in China.
***Acanthocephalus goaensis*** Jain and Gupta, 1981 in India.
***Acanthocephalus gotoi*** Van Cleave, 1925 in Japan.
***Acanthocephalus halongensis*** Amin and Ha, 2011 in Vietnam.
***Acanthocephalus hastae*** Baylis, 1944 in Australia.
***Acanthocephalus japonicus*** (Fukui and Morisita, 1936) Petrochenko 1956 [syns. *Filisoma japonicum* Fukui and Morisita, 1936; *Acanthocephaloides japonicus* (Fukui and Morisita, 1936) Yamaguti, 1939] in Japan.
***Acanthocephalus kabulensis*** Datta and Soota, 1956 in Afghanistan.
***Acanthocephalus kashmirensis*** Datta, 1936 in India.
***Acanthocephalus lizus*** Li-Minmin, 1984 in Japan.
***Acanthocephalus loktakensis*** Shomorendra, Ranibala et Jha, 2009 in India
***Acanthocephalus manipurensis*** Bhattacharya, 2007 in India.
***Acanthocephalus minor*** Yamaguti, 1935 in Japan.
***Acanthocephalus nanus*** Van Cleave, 1925 in Japan.
***Acanthocephalus nickoli*** Khan and Bilqees, 1994 in Pakistan.
***Acanthocephalus opsariichthydis*** Yamaguti, 1935 (*nec opsalichtydis*, *nec opsalichthydis*) (*vide* Yamaguti 1939) in Japan.
***Acanthocephalus parallelotestis*** Achmerov et Dombrovskaja-Achmerova, 1941 in Siberia.
***Acanthocephalus serendibensis*** Crusz and Mills, 1970 in Sri Lanka.
***Acanthocephalus sichuanensis*** Wang and Zhang, 1987 in China.
***Acanthocephalus sinensis*** Van Cleave, 1937 in China and Sulawesi.
***Acanthocephalus srilankensis*** Crusz and Ching, 1976 in Sri Lanka.
***Acanthocephalus tenuirostris*** (Achmerov et Dombrovskaja-Achmerova, 1941) Yamaguti 1963 [syn. *Paracanthocephalus tenuirostris* Achmerov et Dombrovskaja Achmerova, 1941] in Siberia.
***Acanthocephalus tigrinae*** (Shipley, 1903) Yamaguti, 1963 [syn. *Echinorynchus tigrinae* Shipley, 1903] in Thailand.

Of the 26 species listed above, and in addition to A. *criniae*, only *A. curtus* has a near fusiform trunk, the posterior part of *A. nickoli* appears sharply pointed, and the trunk of *A. minor* is sub-cylindrical. All three species, however, have ovoid, round, or pyriform cement glands. Only *A. sinensis* has a proboscis armature that overlaps that of *A. parallelcementglandatus* n. sp., 15–19 longitudinal rows of 4–6 hooks each. The hooks of *A. sinensis* are, however, considerably larger than those of the new species, being 53–94, 53–115 anteriorly, 66–103, 79–115 at middle, and 53–98, 53–98 basally, in males and females, respectively. In addition, *A. sinensis* is a parasite of amphibians, *Pelophylax (=Rana) nigromaculatus* (Hallowell, 1861) and *Bufo japonicus formosus* Boulenger, 1883 in China [34].

## *Pseudoacanthocephalus coniformis* n. sp.

(Figs. 19–22)

urn:lsid:zoobank.org:act:E5164365-3507-48A6-AECE-4A2BFDCA5BD9

Type host: *Hylarana* sp. (Ranidae)

Type locality: Bidoup Nui Ba National Park, Lâm Dông Province in southeast Vietnam (12°18′ N; 108°40′ E), Vietnam.

Type specimen: HWML collection no. 49918 (holotype male).

Etymology: The new species is named for its characteristic cone at the anterior end of the trunk.

One adult male was collected from 1 of 2 frogs, *Hylarana* sp. (Ranidae), in the Bidoup Nui Ba National Park, Lâm Dông Province in southeast Vietnam (12°18′ N; 108°40′ E) on June 6, 2013. The genus *Hylarana* Tschudi, 1838 contains around 86 species found from Sri Lanka to the western Ghats of India, through Nepal and southern China and Taiwan, down to southeast Asia to the Philippines and Papua New Guinea, and in Northern Australia as well as in tropical Africa [31].

### Description

*Male* (based on one mature male with sperm): with characters of the genus *Pseudoacanthocephalus* (Echinorhynchidae). Trunk small, unarmed, cylindrical, 7.40 mm long by 0.95 mm wide. Anterior 1/2 of trunk wider with anterior cone 198 long by 270 wide posteriorly (Fig. 19). Proboscis cylindrical, slightly swollen posteriorly, truncated anteriorly, 988 long by 260 wide posteriorly, with 13 rows of 7 rooted hooks each (Figs. 20, 21). Anterior hooks largest, gradually decrease in size posteriorly. Posterior dorsal hooks relatively longer than ventral. Hook roots prominent, slender blade-like, slightly curved (Fig. 20). Length of dorsal hooks (and roots) from anterior 75 (40), 75 (45), 70 (45), 70 (50), 70 (45), 72 (50), 62 (42). Length of ventral hooks (and roots) from anterior 75 (37), 75 (50), 72 (42), 62 (42), 60 (47), 67 (50), 58 (32). Posteriormost hooks most slender, anteriormost hooks next most slender. Proboscis receptacle double-walled but outer wall not continuous posteriorly where retractor muscles pass (Fig. 19), 988 long by 260 wide, with cephalic ganglion at its posterior end. Lemnisci sub-equal, somewhat shorter and longer than receptacle. Longer lemniscus 1.02 long by 0.13 mm wide posteriorly; shorter lemniscus 0.83 long by 0.15 mm wide. Reproductive system in posterior 3/5 of trunk. Testes oblong, large. Anterior testis 0.97 mm long by 0.56 mm wide. Posterior testis 1.05 mm long by 0.48 mm wide. Cement glands 8, filiform, staggered, 1.56–1.72 (1.64) mm long by 0.11–0.16 mm wide, with long ducts. Each of four cement gland ducts join into one duct; both common ducts join other reproductive ducts into genital orifice. Saefftigen’s pouch 520 long by 125 wide. Saefftigen’s pouch, common sperm duct and common cement gland ducts overlap and join into spheroidal non-ornate bursa, 759 long by 728 wide (Fig. 22).

### Remarks

Amin et al. [9] and Tkach et al. [31] provided keys to the species of *Pseudoacanthocephalus* that emphasized proboscis armature. Later, Amin (2013) listed 18 species, most of which are from Asian or associated geographies. The new species differs from all 18 species listed below in 2 traits, proboscis armature aside. (1) The anterior trunk of *P. coniformis* n. sp. features a prominent cone. A cone at the anterior trunk was not described in any of the 18 listed species. However, Figure 4A, E in Tkach et al. [31] suggest that *Pseudoacanthocephalus smalesi* may have an anterior trunk cone. *Pseudoacanthocephalus smalesi* is distinguished from *P. coniformis* n. sp. by having a small fusiform trunk, 12 proboscis hook rows each with 4–5 hooks, only 4 cement glands, and much larger hooks reaching 108 long, except for the smaller posteriormost hook. (2) The cement gland pattern of *P. coniformis* is uniquely different. All species of *Pseudoacanthocephalus* have six cement glands, as per the generic diagnosis, with three exceptions, *P. coniformis* and *P. nguyenthileae* with eight glands, and *P. smalesi* with four. In *P. coniformis*, the eight cement glands are long, filiform, and staggered longitudinally in a sequence one after the other. The cement gland ducts are also long and hardly marked off the glands. The cement glands of all the other species are usually claviform, with two qualified exceptions. The eight cement glands of *P. nguyenthileae* are filiform to claviform arranged in two overlapping anterior and posterior tiers each with four glands [9]. The six cement glands of *P. reesei* are in one tightly packed cluster at the same level; no staggering [15].

The 18 known species of *Pseudoacanthocephalus* discussed above are listed below:

***Pseudoacanthocephalus betsileo*** Golvan, Houin et Bygoo, 1969 in Madagascar.
***Pseudoacanthocephalus*** *s* ***bigueti*** (Houin, Golvan et Bygoo, 1965) Golvan, 1969 [syn. *Acanthocephalus bigueti* Houin, Golvan et Bygoo, 1965] in Madagascar.
***Pseudoacanthocephalus bufonicola*** (Kostylew, 1941) Petrochenko, 1956 [syn. *Acanthocephalus bufonicola* Kostylew, 1941] (*nec bufonincola*) in Central Asia and Eastern Europe.
***Pseudoacanthocephalus bufonis*** (Shipley, 1903) Petrochenko, 1956 (**type species**) [syns. *Echinorhynchus bufonis* Shipley, 1903; *Acanthocephalus bufonis* (Shipley, 1903) Southwell et MacFie, 1925 sensu Petrochenko, 1953; *A. breviprostatus* Kennedy, 1982; *A. sinensis* Van Cleave, 1937] in Thailand.
***Pseudoacanthocephalus caspanensis*** (Fernández et Ibarra Vidal, 1992) Arredondo et Gil de Pertierra, 2009 [syn. *Acanthocephalus caspanensis* Fernández et Ibarra Vidal, 1992]. In the Andean mounains of South America, Chile and Paraguay (?).
***Pseudoacanthocephalus caucasicus*** (Petrochenko, 1953) Petrochenko, 1956 [syn. *Acanthocephalus caucasicus* Petrochenko, 1953] in Central and Eastern Europe.
***Pseudoacanthocephalus elongatus*** (Van Cleave, 1937) Petrochenko, 1958 in Hunan, China.
***Pseudoacanthocephalus lutzi*** (Hamann, 1891) Arredondo et Gil de Pertierra, 2009 [syns. *Echinorhynchus lutzi* Hamann, 1891; *Acanthocephalus lutzi* (Hamann, 1891) Meyer, 1932; *Acanthocephalus saopaulensis* Smales, 2007; *Pseudoacanthocephalus saopaulensis* (Smales, 2007) Arredondo et Gil de Pertierra, 2009] in Argentina and Brazil.
***Pseudoacanthocephalus nguyenthileae*** Amin, Ha et Heckmann, 2008 in Vietnam.
***Pseudoacanthocephalus nickoli*** Tkach, Lisitsyna, Crossley, Binh et Bush, 2013 in the Philippines.
***Pseudoacanthocephalus paratiensis*** Bhattacharya, 2000 in India.
***Pseudoacanthocephalus perthensis*** Edmonds, 1971 in Australia.
***Pseudoacanthocephalus rauschi*** Gupta et Fatma, 1986 in India.
***Pseudoacanthocephalus reesei*** Bush, Duzynski et Nickol, 2009 in China.
***Pseudoacanthocephalus rhampholeonotos*** Smales, 2005 in Tanzania.
***Pseudoacanthocephalus shillongensis*** Bhattacharya, 1999 in India.
***Pseudoacanthocephalus smalesi*** Tkach, Lisitsyna, Crossley, Binh et Bush, 2013 in the Philippines.
***Pseudoacanthocephalus xenopeltidis*** (Shipley, 1903) Golvan, 1969 [syn. *Echinorhynchus xenopeltidis* Shipley, 1903] in Thailand.

The cement gland pattern could not be discerned in *P. elongates* and *P. paratiensis* as their descriptions were based on females only; no trunk cones were described or illustrated. Not much information is available on the contracted 15–25-mm-long specimens of *P. xenopeltidis* except for having 8–12 proboscis hook rows in a dissected invaginated proboscis [19].

## *Cathayacanthus spinitruncatus* n. sp.

(Figs. 23–44)

urn:lsid:zoobank.org:act:C81AFB27-5691-4660-97B3-0001FB67AF4C

Type host: The common pony fish, *Leiognathus equulus* (Leiognathidae).

Type locality: The Hue City area south of Tonkin Gulf, Thua Thien Hue Province, Central Vietnam (16°43′ N; 107°45′ E), Vietnam.

Type specimen: HWML collection no. 49919 (holotype female), no. 49920 (paratype female).

Etymology: The new species is named for its fully spined trunk.

Six adult females were collected from 4 of 5 common pony fish, *Leiognathus equulus*, south of Tonkin Gulf at the Hue City area, Thua Thien Hue Province, Central Vietnam (16°43′ N; 107°45′ E) in August, 2013. Two specimens were used for SEM studies. The common pony fish is a widely distributed Indo-Pacific species found in East Africa, the Red Sea, the coast of India, throughout southeast Asia to southern Japan and northern Australia, and eastwards to Samoa [21]. It occurs in inshore muddy-bottomed coastal waters, river mouths and estuaries, sometimes entering lower reaches of freshwater streams [1]. It feeds on polychaetes, small fishes, and crustaceans [35].

Golvan’s [19] relegation of *Rhadinorhynchus exilis* Van Cleave, 1928 from the Crucian carp, *Carassius carassius* (Linn.) (Cyprinidae), in China to his new genus *Cathayacanthus* based on the absence of large specialized basal proboscis hooks was perfectly justified. Furthermore, the cephalic ganglion of *C. exilis* appears to be at the anteriormost end of the proboscis receptacle, the same as in our specimens. Van Cleave’s [33] very brief and incomplete description made no reference to the cephalic ganglion but his Figure 9 clearly showed the ganglion’s anterior position. The cephalic ganglion in *Rhadinorhynchus* is near the middle of the proboscis receptacle. Van Cleave [33] also stated “hooks on the ventral surface conspicuously larger than those on dorsal surface and more strongly recurved” as shown in his Figure 9. Our specimens show the same dorso-ventral differentiation of proboscis hooks.

The only other species of *Cathayacanthus* is *C. bagarri* Moravec and Sey, 1989, which was collected from another freshwater fish, *Bagarius bagarius* (Hamilton) (Sisoridae), in the Red River near Hanoi, Vietnam. The anterior end of all described specimens of *C. bagarri* was withdrawn with the proboscis totally inverted within the receptacle. Moravec and Sey [27] dismissed the importance of “alleged dorsoventral asymmetry of proboscis hooks” advanced by Golvan [19] and included in Van Cleave’s [33] (1928) treatment because they could not demonstrate it in their distorted specimens. They also indicated and showed (Fig. 6a, h) the position of the cephalic ganglion to be in the “posterior half” of the receptacle. Those two characters alone conflict with their assignment of their specimens to *Cathayacanthus*, which was based on the absence of specialized basal proboscis hooks. We assume that the cephalic ganglion was displaced posteriorly by the invaginated proboscis in the withdrawn receptacle, which also obscured the delineation of dorsoventral hook asymmetry. That species is provisionally retained in *Cathayacanthus* as per Amin [2] until more informative specimens are examined.

*Cathayacanthus spinitruncatus* n. sp. is distinguished from the above two species by having a very long and slender proboscis with more than 50 hooks per row and a totally spined trunk. The proboscis of *C. exilis* and C. *bagarri* has 12 longitudinal rows of 32 proboscis hooks each and 14 rows of 33–35 hooks each, respectively; both species have only small anterior trunk spines. The anterior trunk spines of *C. spinitruncatus* are large and the proboscis has 14 longitudinal rows of 53–61 hooks each. The proboscis of *C. spinitruncatus* is sharply curved dorsally, similar to that of *C. exilis* (Fig. 9 of Van Cleave) [33].

*Rhadinorhynchus plagioscionis* Thatcher, 1980 collected from *Plagioscionis squamosissimus* (Heckel) in the Brazilian Amazon poses a different kind of taxonomic problem. The proboscis of that species also possesses no basal specialized hooks and would qualify as another species of *Cathayacthus.* Proboscis hooks are also dorsoventrally asymmetrical and become considerably smaller and more crowded posteriorly, as is the case in *C. spinitruncatus*. Its trunk is almost totally armed with small spines except posteriorly. However, its cephalic ganglion was clearly described and shown (Fig. 6) to be in the posterior half of the receptacle and not at its anteriormost end, as is characteristic of *Cathayacanthus.* It is also found in the “wrong” continent.

### Diagnosis of the genus *Cathayacanthus*

Given the above information and that in Moravec and Sey [27] cautiously interpreted, Golvan’s [19] diagnosis of the genus *Cathayacanthus* should be modified (changes in bold) to read: “Rhadinorhynchinae, parasites of freshwater fish in **East Asia**. Cuticular trunk spines very small **or enlarged anteriorly**, in one anterior zone **or covering whole trunk**. Proboscis long, subcylindrical, armed with hooks showing dorsoventral asymmetry, decrease in size **and crowd** posteriorly, **with no large specialized basal hooks. Proboscis receptacle double-walled, markedly longer than proboscis, with cephalic ganglion at anteriormost end. Cement glands 4, tubular. Female gonopore terminal**. **Eggs spindle-shaped with polar prolongation of fertilization membrane**.”

### Description

**Figures 23–30:**
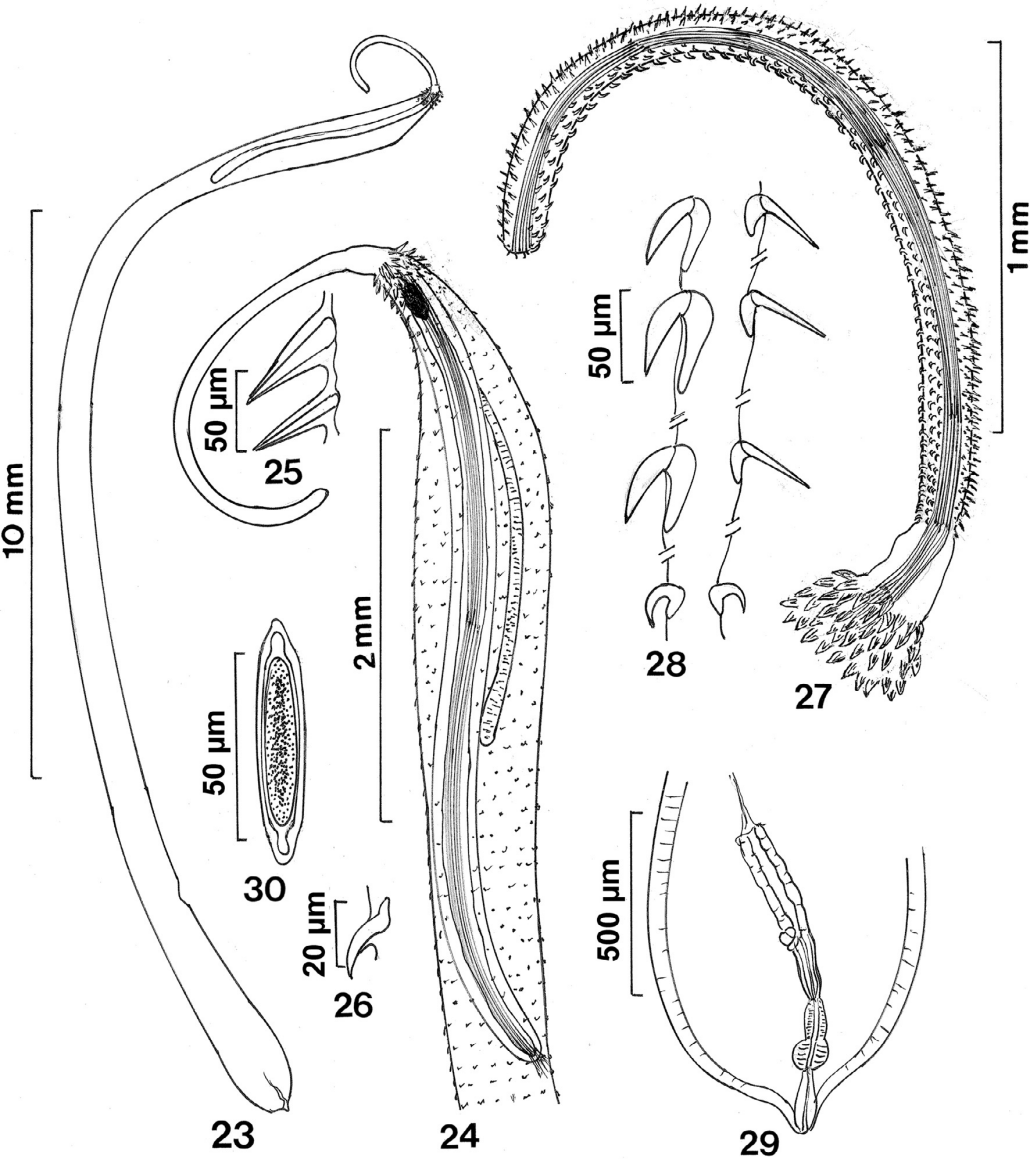
*Cathayacanthus spinitruncatus* n. sp. from *Leiognathus equulus.* 23. Holotype female. 24. Anterior end of specimen in Figure 23. Note the very long receptacle, the strong retractor muscles, the spiny trunk, and the anterior position of the cephalic ganglion. 25. The first and the second of the anterior cluster of trunk spines. 26. A ventral trunk spine near the middle of the trunk. 27. The proboscis, neck and part of the anterior cluster of trunk spines. Note the strong retractor muscles. Hooks overlapping retractor muscles are not shown. 28. Hooks nos. 1, 5, 15, 60 from anterior in ventral (left) and dorsal (right) rows. Note the extreme dorso-ventral diversification; lateral hooks are intermediate. 29. Reproductive system. 30. Egg.

**Figures 31–36:**
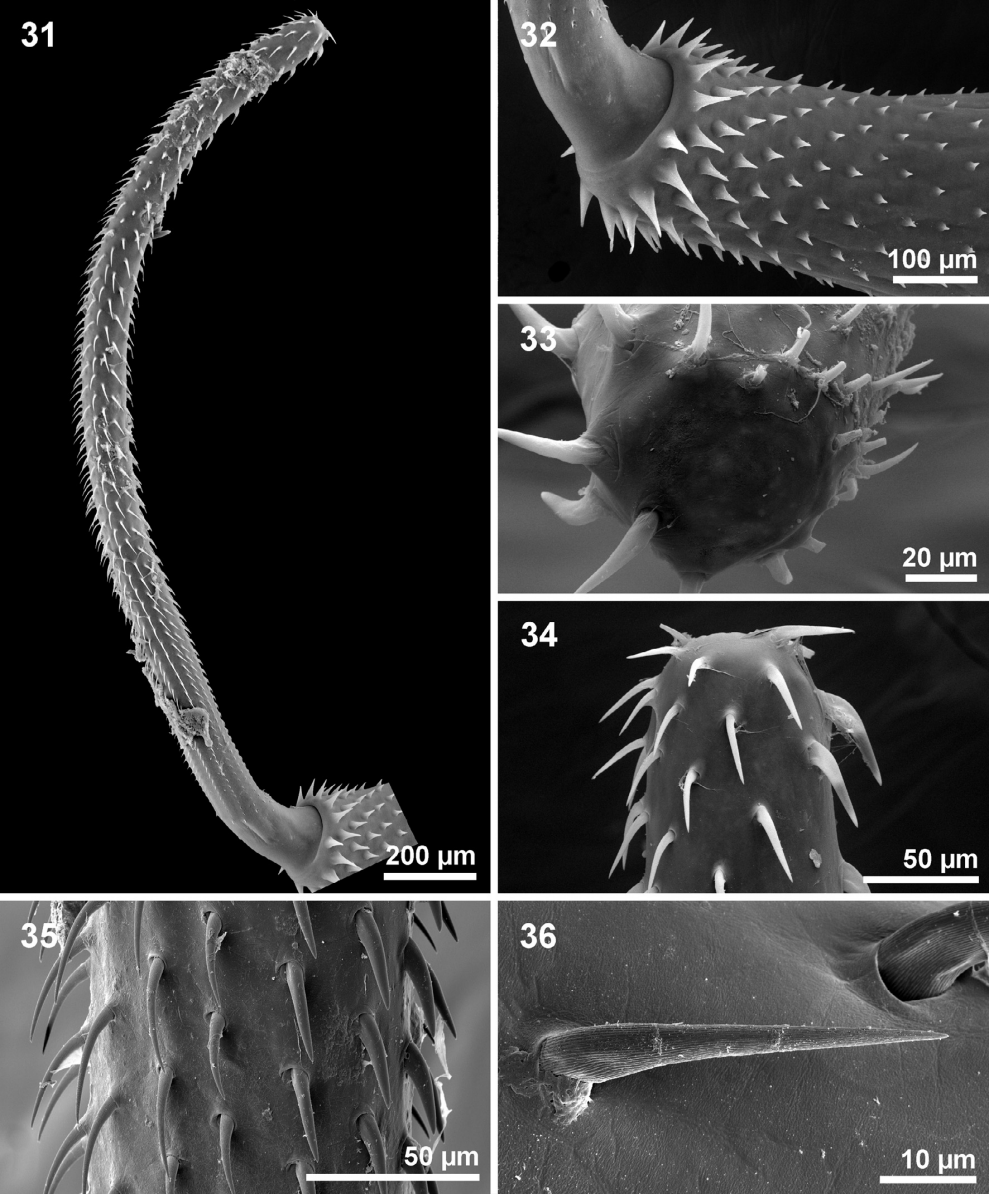
SEM of *Cathayacanthus spinitruncatus* n. sp. from *Leiognathus equulus.* 31. Fully everted proboscis of a paratype female. 32. The long neck and anterior trunk of the same specimen in Figure 31 showing the sharply decreasing size of trunk spines. 33. The bald apical end of the proboscis in one specimen. 34. A lateral view of the anterior end of the proboscis of a paratype female showing the differentiation between the ventral hooks (right) and dorsal hooks (left). 35. The differentiation between ventral hooks (right) and dorsal hooks (left) at the middle of the proboscis of the same specimen in Figure 34. 36. A close-up of hooks showing their longitudinally ribbed surface.

**Figure 37–44:**
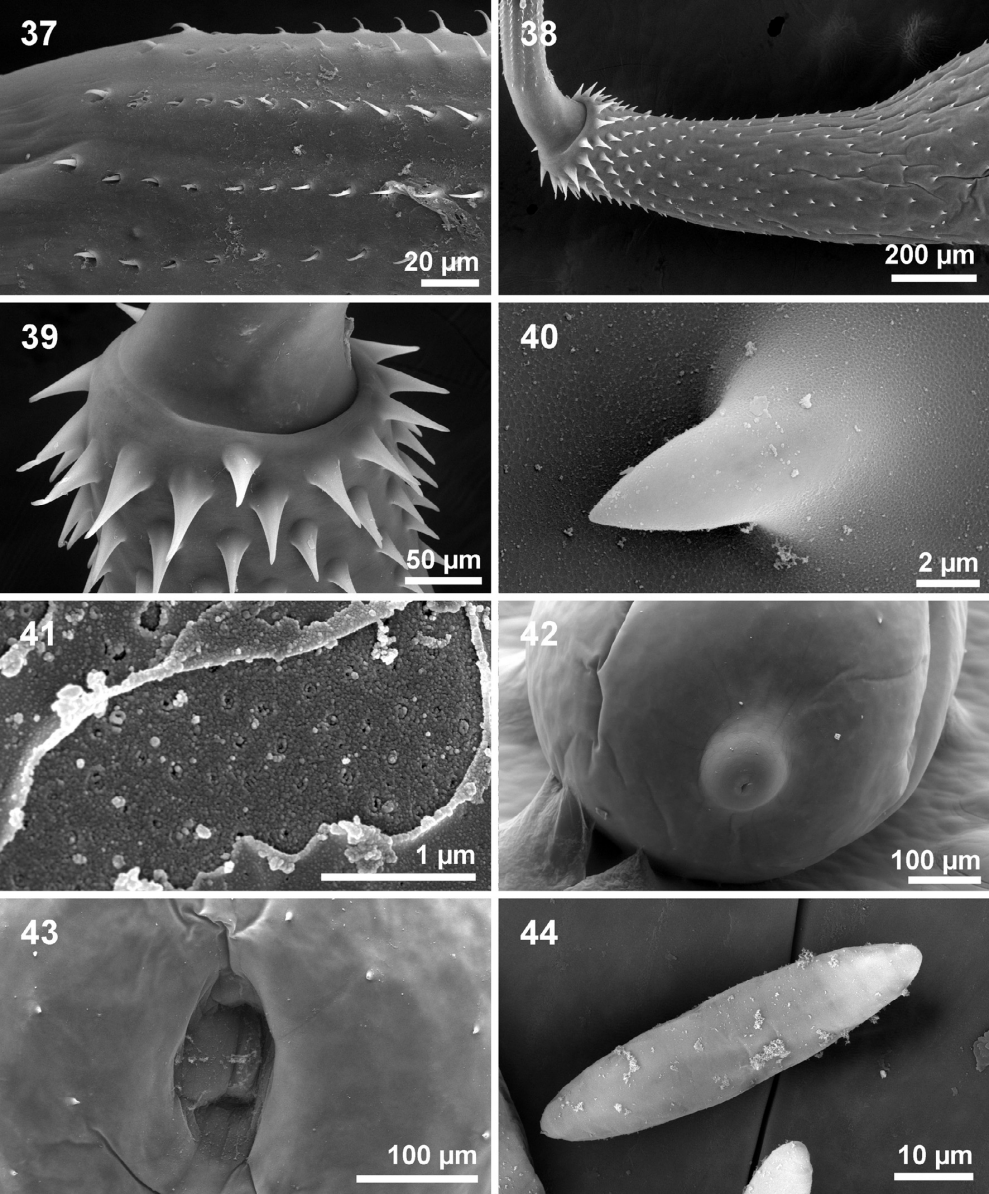
SEM of *Cathayacanthus spinitruncatus* n. sp. from *Leiognathus equulus.* 37. The markedly decreasing size of posterior hooks and the slight increase in the size of the basal hook in the proboscis of a paratype female. 38. The anterior trunk of a paratype female showing the progressively wider spacing of posterior trunk spines compared with the anterior spines. 39. The enlarged anterior trunk spines and the clear separation of the trunk from the neck in one specimen. 40. A high magnification of a trunk spine at the middle trunk. 41. An area of the mid-trunk cleared from encrusted film of sediment showing the micropores. 42. The posterior end of a female specimen showing its nipple shape. 43. The female genital orifice showing the extension of trunk spines to the posteriormost end of the trunk. 44. A ripe egg.

Females (based on three gravid adults and one specimen with ovarian balls): Rhadinorhynchinae. Specimens long, cylindrical, slender, arched. Trunk 14.27–23.07 (18.46) mm long by 0.62–0.76 (0.71) wide anteriorly and 0.60–1.17 wide posteriorly. Trunk wholly spined; spines with no dorso-ventral differentiation (Figs. 23, 24, 32, 38–40, 43). Anterior spines largest in about 6 rings of 21–24 spines each, in posteriorly decreasing size and in alternating or longitudinal rows (Figs. 25, 27, 32, 38, 39). Length of large spines from anterior 55–65 (61), 50–65 (55), 37–45 (41), 31–37 (34), 29–37 (33), 25–32 (28). Remaining spines (Figs. 26, 40, 43) on whole trunk to posterior extremity in many rings of 7–10 spines on each side, 20–27 (23) long except most posterior spines being 15–17 (16) long (Fig. 24). Proboscis long, slender, curved dorsally (Fig. 31), widest at posterior end, 2.25–2.60 (2.38) mm long by 0.14–0.17 wide posteriorly, with one lateral pair of sensory pits between 2 and 3 posteriormost hooks. All proboscis hooks with ribbed surface (Fig. 36), rooted, in 14 longitudinal rows with 53–61(in two specimens) hooks each. Hooks dorsoventrally asymmetrical with ventral and lateroventral hooks more robust and strongly recurved than slender and straight dorsal and laterodorsal hooks (Figs. 28, 34, 35). Shape of ventral and dorsal hooks’ transition gradually being intermediate laterally (Figs. 34, 35). Hooks largest in anterior half of proboscis and gradually becoming smaller and more crowded posteriorly; posterior 10 hooks smallest and most crowded. No large specialized basal hooks. Basal hooks, however, slightly larger than pre-basal hooks (Fig. 37). Length and width (at base) of every 5 ventral hooks numbered from anterior: (hook # 1) 40–45 (42) × 12–15 (13), (# 5) 52 (52) × 19–20 (19), (# 10) 52 (52) × 20 (20), (# 15) 50–52 (51) × 20 (20), (# 20) 47–49 (48) × 20 (20), (# 25) 432–47 (44) × 17–20 (18), (# 30) 40–42 (41) × 17 (17), (# 35) 30–35 (32) × 14–15 (14), (# 40) 22–27 (24) × 10–11 (10), (# 45) 21–22 (21) × 10 (10), (# 50) 20 (20) × 10–11 (10) (10), (# 55) 15–17 (16) × 9–11 (10), (# 60) 15–17 (16) × 10 (10), (# 61, basal) 20 (20) × 10–11 (10). Length and width (at base) of every five dorsal hooks numbered from anterior: (hook # 1) 35–40 (37) × 4–5 (4), (# 5) 35–47 (41) × 5–6 (5), (# 10) 50 (50) × 5–6 (5), (# 15) 49–52 (51) × 5–6 (5), (# 20) 49–52 (50) × 5–6 (5), (# 25) 42–50 (46) × 5–6 (5), (# 30) 42–47 (45) × 5–7 (6), (# 35) 42–47 (45) × 5 (5), (# 40) 37–47 (42) × 5 (5), (# 45) 30–43 (36) × 4–5 (5), (# 50) 22–37 (29) × 4–5 (4), (# 55) 19–27 (23) × 4–5 (4), (# 60) 15–16 (15) × 3–4 (3), (# 61, basal) 15–19 (17) × 7 (7). Hook roots simple, directed posteriorly, proportional in size to length of blades (Fig. 28). Roots of ventral hooks slightly shorter than blades. Roots of dorsal hooks markedly shorter than blades. Roots are intermediate in lateral hooks. Neck 281–312 (291) long by 198–229 (208) wide posteriorly. Proboscis receptacle markedly longer than proboscis, double-walled, with cephalic ganglion at its anteriormost end. Lemnisci multinucleated, subequal, long, but shorter than receptacle, cylindrical, gradually tapering posteriorly (Fig. 24). Reproductive system very short compared with trunk length, 832 long in one specimen (5% of trunk length), with vagina lacking terminal sphincter, constricted uterus, and short uterine bell with few nucleated cells. Posterior end rounded with terminal gonopore in papillated orifice (Figs. 29, 42, 43). Eggs oblong, 62–67 (65) long by 12–17 (15) wide, with polar prolongation of fertilization membrane (Figs. 30, 44).

## *Rhadinorhynchus johnstoni* Golvan, 1969

One female specimen in the ovarian ball stage was collected from the marine darkbar flying fish *Cypselurus hexazona* (Exocoetidae) in the Pacific Ocean at Quang Binh Province along Vietnam’s north central coast south of Tonkin Bay (17°31′ N; 106°39′ E) on April 24, 2013. The host, *C. hexazona*, is reported in the Indo-West Pacific, Red Sea to the Philippines, Vietnam, New Guinea and New South Wales, Australia. It is found in near-shore surface waters, never spread to open sea, and feeds on zooplankton and small fish [28, 29].

### Description of our Vietnamese specimen

*Female* (based on one specimen in the ovarian ball stage): with characters of the genus Rhadinorhynchus (Rhadinorhynchidae). Specimen long, cylindrical, with anterior half somewhat wider and gradually merging with posterior half, 13.55 mm long by 0.57 mm wide anteriorly. Anterior cuticular trunk spines in two regions barely separated by spine-free zone. Anterior group of trunk spines completely encircling trunk, in 3–4 loosely arranged rings with 12–14 spines each. Anterior spines 47 long, posterior spines 57 long. Second group of spines in 14 more loosely arranged rings of dorsally incomplete rings of spines; dorso-lateral spines disappear progressively posteriorly, ending with one posteriormost spine. Ventral spines somewhat longer than dorsal spines of second group: dorsal spines 59 long anteriorly and 67 long posteriorly; ventral spines 62 long anteriorly and 82 long posteriorly. Cuticular spines deltoid-shaped, with the largest being about as long as the longest proboscis hook (82 long). Proboscis long, 1.87 mm long by 0.23 mm wide, with 17 rows of 25 hooks each. Length of hooks 45, 75, 82, 75, 47, 80 in apical, sub-apical, middle, posterior, posteriormost and basal ring positions, respectively. Neck 385 long by 260 wide posteriorly. Proboscis receptacle about twice as long as proboscis, 3.57 mm long by 0.27 mm wide. Reproductive system long, about 1/3 as long as trunk: 4.38 mm long; 32% of body length. Length of uterine bell, very long uterus and vagina 0.24 mm, 3.94 mm, and 0.20 mm, respectively. Posterior end of trunk obtuse with subterminal gonopore.

### Remarks

One immature female specimen of *R. johnstoni* was poorly described as *R. pristis* (Rudolphi, 1802) Lühe, 1911 by Johnston and Edmonds [22]; it was collected from the intestine of a southern bluefin tuna, *Thunnus maccoyi* (Castelnau, 1872), caught in St. Vincent Gulf, South Australia. The worm was 17.1 mm long with an invaginated proboscis (1.9 mm long), a proboscis receptacle (2.8 mm long ?) and unripe eggs (62 × 12). Johnston and Edmonds [22] made reference to the two groups of trunk spines and illustrated the anterior end of their specimen, two trunk spines and the egg. Golvan [19] repeated Johnston and Edmonds’s [22] description and assigned it to the genus *Rhadinorhynchus* as *R. johnstoni*. That species was not reported until Amin and Nahhas [5] provided for the first time a full description of a few specimens of the species collected from a related fish, the mackerel tuna, *Euthynnus affinis* (Cantor, 1849) off the Fiji Islands. The host of our specimen from Vietnam, *C. hexazon*, belongs to a distant family of flyfishes, Exocoetidae [Actinopterygii (ray-finned fishes): Beloniformes (needle fishes)], while *T. maccoyi* and *E. affinis* belong to the Family Scombridae [Actinopterygii (ray-finned fishes): Perciformes], suggesting a wide adaptability to a diverse host taxa.

Specimen: HWML collection no. 49921.
